# Scalable Bayesian phylogenetics

**DOI:** 10.1098/rstb.2021.0242

**Published:** 2022-10-10

**Authors:** Alexander A. Fisher, Gabriel W. Hassler, Xiang Ji, Guy Baele, Marc A. Suchard, Philippe Lemey

**Affiliations:** ^1^ Department of Statistical Science, Duke University, Durham, NC 27710, USA; ^2^ Department of Computational Medicine, David Geffen School of Medicine at UCLA, University of California, Los Angeles, CA 90095, USA; ^3^ Department of Biostatistics, Jonathan and Karin Fielding School of Public Health, University of California, Los Angeles, CA 90095, USA; ^4^ Department of Human Genetics, David Geffen School of Medicine at UCLA, University of California, Los Angeles, CA 90095, USA; ^5^ Department of Mathematics, School of Science and Engineering, Tulane University, New Orleans, LA 70118, USA; ^6^ Department of Microbiology, Immunology and Transplantation, Rega Institute, KU Leuven, 3000 Leuven, Belgium

**Keywords:** Bayesian phylogenetics, scalable inference, online inference, Hamiltonian Monte Carlo, BEAST, adapative MCMC

## Abstract

Recent advances in Bayesian phylogenetics offer substantial computational savings to accommodate increased genomic sampling that challenges traditional inference methods. In this review, we begin with a brief summary of the Bayesian phylogenetic framework, and then conceptualize a variety of methods to improve posterior approximations via Markov chain Monte Carlo (MCMC) sampling. Specifically, we discuss methods to improve the speed of likelihood calculations, reduce MCMC burn-in, and generate better MCMC proposals. We apply several of these techniques to study the evolution of HIV virulence along a 1536-tip phylogeny and estimate the internal node heights of a 1000-tip SARS-CoV-2 phylogenetic tree in order to illustrate the speed-up of such analyses using current state-of-the-art approaches. We conclude our review with a discussion of promising alternatives to MCMC that approximate the phylogenetic posterior.

This article is part of a discussion meeting issue ‘Genomic population structures of microbial pathogens’.

## 1. Introduction

Pathogen genomic data have become an invaluable resource to study microbial evolution and infectious disease transmission. The widespread adoption of whole-genome sequencing technologies ensures an ever-increasing scale at which genomic data have become available. EnteroBase for example offers over 300 000 assembled genomes for several bacterial genera and facilitates the genotyping and characterisation of novel strains against all natural populations [1]. Rapidly expanding genome sequencing efforts are being undertaken in response to every new viral epidemic. Over 1500 Ebolavirus genomes were generated during the 2013–2016 West-African Ebola epidemic, representing over 5% of the known cases [2]. At the time, this was considered as a new scale in viral genomic monitoring, and the adoption of portable genome sequencing also offered the perspective of providing it in close to real time [3]. Global sequencing efforts during the COVID-19 pandemic have again taken genomic epidemiology to new heights, with over 4 000 000 SARS-CoV-2 genomes available in GISAID at the time of writing [4].

The potential contribution of genomic epidemiology to the public health response during outbreaks and epidemics hinges on the ability to provide insights in short turnaround times. This has motivated the development of analytic and visualization tools such as Nextstrain for real-time tracking of pathogen evolution and spread [5]. However, many of the phylodynamic models that rely on phylogenies to unravel pathogen transmission dynamics have traditionally been implemented in Bayesian inference frameworks. A Bayesian approach accommodates phylogenetic uncertainty in the phylodynamic estimates and offers extensive modelling flexibility, but represents a computationally challenging endeavour as it requires averaging over all plausible evolutionary histories. This becomes particularly problematic when confronting data encompassing thousands of genomes. The COVID-19 genomic response has therefore demonstrated the pressing need for more scalable phylodynamic inference methodologies.

In this review, we present recent developments in Bayesian phylogenetics that begin to answer this call for scalable phylodynamic methods. In addition to offering a unified and coherent framework to quantify uncertainty in phylogenetic parameters, the Bayesian approach allows one to learn the joint distribution of all phylogenetic parameters simultaneously. This is particularly important when combining the phylogenetic model with molecular clock models, tree generative models and trait evolutionary models, which is the focus of the BEAST Bayesian inference framework [6,7]. Here we briefly review this Bayesian phylogenetic framework to guide further exposition. For an introductory review of Bayesian phylogenetics, see Nascimento *et al.* [8].

Within the Bayesian phylogenetic setting, one chooses a data generative model to describe the evolution of genetic sequence data, phenotypic traits, the geographic displacement of sampled taxa and more. This data generative model is referred to as the *likelihood*. Subsequently, one places a *prior* distribution on parameters of the likelihood that describe a plausible range of values these parameters may take. Next, one estimates the *posterior* distribution of parameters of interest, typically via Markov chain Monte Carlo (MCMC) integration, the main workhorse of Bayesian phylogenetic inference. To begin MCMC sampling, initialize the Markov chain by setting a possible state for each unknown model parameter. MCMC sampling proceeds by iterating over two steps. First, propose a new state where one or more parameters are updated and compare the posterior density of the new state with the old. Next, accept the new state if the corresponding posterior is higher but occasionally accept the new state if the posterior is lower. See Van Ravenzwaaij *et al.* [9] for an introduction to MCMC. For the purpose of this review, we classify the current limitations of inference as follows:

1. every step in MCMC requires evaluating the posterior ratio which in turn requires a potentially computationally demanding *likelihood* calculation;
2. tree-space can be enormous, requiring many steps before the MCMC algorithm is able to sample from the posterior (the *burn-in* period);
3. standard MCMC proposals only allow one to make small steps, limiting the efficiency by which samples from the posterior can be obtained.

In order to maximize the amount of information that can be obtained about the posterior per unit of time, scalable machinery largely focuses on directly improving MCMC by speeding up likelihood calculation, reducing burn-in, or developing more efficient proposals as well as indirectly improving MCMC by constructing models amenable to scalable MCMC techniques.

The first challenge, slow likelihood computation, is a burden shared by various phylogenetic inference approaches and this has motivated the development of libraries for optimized likelihood evaluation [10,11]. The BEAST inference framework relies on the BEAGLE high-performance general-purpose library (https://github.com/beagle-dev/beagle-lib) and we refer interested readers to Ayres *et al.* [11] and Baele *et al.* [12] for further details. From a practical perspective, it is important to note that BEAGLE implements parallelism in the likelihood calculation offering significant speed-ups on modern graphics processing units (GPUs) and multicore central processing units (CPUs). In the remainder of this paper, we turn our attention to inference machinery that reduces burn-in and generates more efficient proposals, and finally conclude with a discussion of novel methods alternative to MCMC for posterior estimation.

## 2. Reducing burn-in via ‘online’ inference

Bayesian phylogenetic inference via MCMC often requires days or weeks of runtime to obtain reasonable estimates of parameters of interest. The addition of new observations extends runtime further by requiring one to restart their MCMC chain as new data become available. Additionally, as the number of taxa in an analysis grows, so does the dimension of parameter space one must explore. For example, the number of possible evolutionary trees grows exponentially with the number of taxa under study. As the size of parameter space grows, it takes longer for the chain to reach regions of high posterior density. This period of searching for the posterior is called the ‘burn-in’ period of the chain. The need to update analyses as new data accumulate is a scenario that now typically arises with real-time genomic responses to viral outbreaks. Sequential analyses associated with growing parameter spaces would therefore benefit from initiating the chain from an informed starting point. Gill *et al.* [13] developed a principled distance-based procedure to add new taxa to an existing tree and install previous MCMC chain samples into a new chain thus allowing one to incorporate new data in an ‘online’ fashion. The approach of Gill *et al.* [13] is publicly available in the BEAST software package. Lemey *et al.* [14] employ this online framework to study geographic spread of SARS-CoV-2 in near real time, adding viral genomes as they became available.

**Figure 1.**
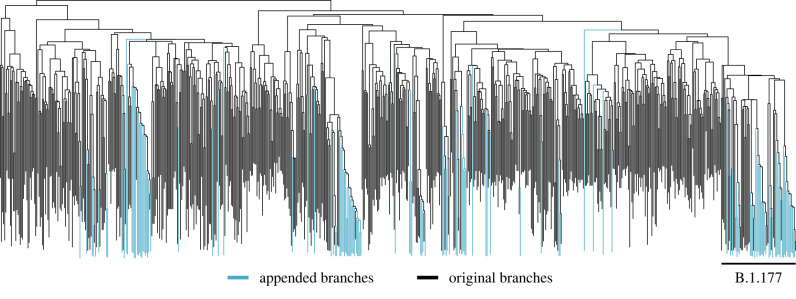
The online addition of 132 SARS-CoV-2 sequences to a 588-tip time-measured tree drawn from the posterior. Appended branches are blue while original branches are black. We omit timescale since this augmented tree is not sampled from the posterior.

We illustrate the online procedure of Gill *et al.* [13] by reconstructing the evolutionary history of 588 full SARS-CoV-2 genomes (29 409 nt) collected from 30 January to 30 September 2020. We follow Lemey *et al.* [14] and use an HKY85 model with among-site rate heterogeneity modelled using a four-category discretised Γ distribution, and a coalescent tree prior. In figure 1, we augment the most recent MCMC sample of our 588-tip tree to include 132 new SARS-CoV-2 sequences sampled between 1 October and 15 October 2020. This procedure takes seconds (less than 1 min) to complete. Notice that all added sequences that belong to the B.1.177 variant, which rapidly spread from Spain to many other European countries over the summer [15], correctly form a monophyly before a single MCMC step is taken with the new 720-tipped tree. As a result, the new, online chain initializes with a log joint density evaluation of −79 768. In comparison, across five runs from five different naive starting trees with 720 tips, it took an average of 6.4 h to sample a joint density evaluation at least as favourable.

## 3. Constructing new chains via better Markov chain Monte Carlo proposals

### (a) Adaptive Markov chain Monte Carlo

As developments in sequencing technology increase both the number and length of genetic sequences available to researchers, it is standard and often appropriate practice to partition long genetic sequences into smaller components (perhaps into individual genes) and assume each partition has its own, independent evolutionary model [16]. This partitioning of the data results in a more flexible and realistic statistical model but dramatically expands the number of parameters to be inferred, as it associates each individual partition with its own set of parameters.

Classical MCMC update procedures, such as univariable Metropolis–Hastings (uMH) update individual parameters sequentially (rather than simultaneously). Univariable samplers are poorly suited for this high-dimensional scenario, as the number of potentially costly likelihood evaluations required to update all parameters scales with the total number of parameters. Similarly, simultaneously updating all parameters (or large blocks of parameters) independently in a single MCMC step typically has extremely low acceptance probability since many parameters have strong and complex posterior relationships that such independent proposals ignore.

Adaptive MCMC (e.g. [17]) addresses this issue by updating all parameters (or large blocks of parameters) simultaneously in a way that accounts for their posterior correlations. Specifically, adaptive MCMC uses the history of the Markov chain itself to approximate posterior covariance between relevant parameters and uses that covariance to propose correlated, rather than independent, parameter updates. Owing to this feature, the adaptive kernel is not Markovian but the resulting chain is still valid [18,19]. As the chain progresses forward, the adaptive MCMC procedure can continuously re-evaluate the posterior covariance and tune relevant hyper-parameters to maximize sampling efficiency.

Baele *et al.* [20] adapt this procedure to Bayesian phylogenetics in the BEAST framework and demonstrate an order of magnitude increase in sampling efficiency for partitioned datasets. While the adaptive procedure is not limited to partition parameters, Baele *et al.* [20] exploit the synergistic interaction between adaptive MCMC and parallel computation in partitioned models. In multi-processor computing environments, each model partition is typically assigned to a single processor. When performing uMH to update a parameter in a partitioned model, only the processor assigned to that partition recomputes the likelihood while the others remain idle. However, when all parameters are updated simultaneously, all processors are simultaneously engaged. As such, while a single proposal in adaptive MCMC requires likelihood calculations over numerous partitions, these calculations require considerably less time than the same number of calculations performed sequentially.

### (b) Hamiltonian Monte Carlo

Like adaptive MCMC, Hamiltonian Monte Carlo (HMC; [21]) can propose updates to many parameters simultaneously with high acceptance probabilities. The key difference is that adaptive MCMC learns about the properties of the posterior empirically (i.e. it relies only on the past history of the Markov chain), while HMC uses the analytic properties of the posterior distribution.

Conceptually, HMC treats the state of model parameters as the position of a particle in a landscape informed by the posterior. At each step in the Markov chain this ‘particle’ (the parameters) is kicked in a random direction and allowed to traverse this posterior-informed landscape according to Hamiltonian dynamics (imagine a hockey puck moving around an uneven, frictionless surface). The position of this particle after a pre-defined amount of time is the parameter proposal.

If the trajectory through the posterior landscape is calculated exactly, then the parameter proposal is accepted with 100% probability. However, this continuous, nonlinear trajectory cannot typically be calculated analytically and must be approximated by a series of discrete, linear steps through the parameter space [22]. In general, taking many small steps results in a better approximation (and therefore higher acceptance probability) than fewer, larger steps. However, each of these discrete steps requires evaluating the derivative or gradient of the log-posterior with respect to the parameters of interest, which is typically computationally rate-limiting. As such, efficiently exploring the posterior via HMC requires balancing the computational cost of the gradient calculations with the size of the discrete steps. Fortunately, methods such as the no-U-turn sampler [23] automatically tune these hyper-parameters and have found success in Bayesian phylogenetic applications, such as for inference of high-dimensional trait correlation [24].

**Figure 2.**
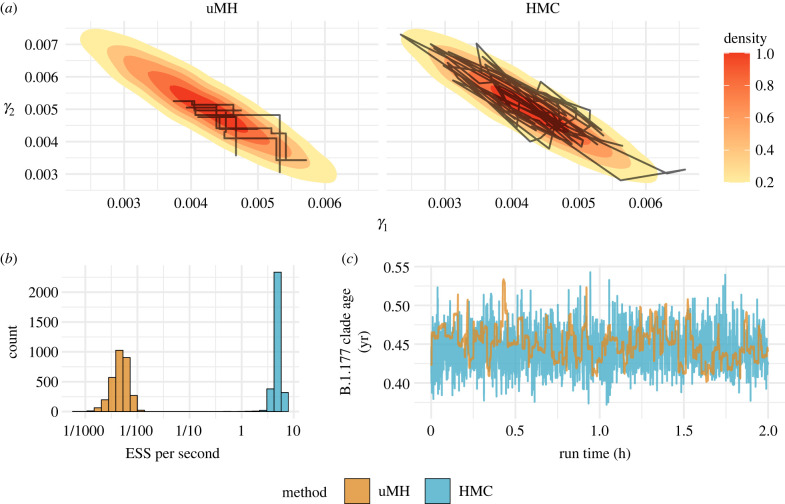
(*a*) Joint trajectory of two branch-specific clock rates *γ*_1_ and *γ*_2_ over their joint density in a three taxa tree with simulated sequence data. Trajectories display 600 posterior samples from the uMH chain and 100 posterior samples from the HMC chain since uMH takes six times as many steps when controlling for runtime. The strong posterior correlation between *γ*_1_ and *γ*_2_ results in very poor mixing with uMH while HMC easily accommodates. (*b*) Effective sample size (ESS) per second of BEAST runtime under both HMC and uMH MCMC samplers of branch-specific rates of phenotypic evolution over a 1536-tip HIV-1 tree. HMC results in a median speed-up of ×1000. (*c*) Trace plot of B.1.177 clade age with node heights sampled under both uMH MCMC and HMC.

We showcase the computational efficiency of HMC against the standard uMH in figure 2. Figure 2*a* displays the trajectory of two clock rate parameters *γ*_1_ and *γ*_2_ over their joint posterior using both HMC and uMH for equivalent amounts of BEAST runtime. For this demonstration, we simulate 12 000 nt of sequence data on a fixed tree with three tips and two different local clocks, *γ*_1_ = 0.003 and *γ*_2_ = 0.006. We choose a three-tip tree since it is the smallest example with identifiable clock structure. We estimate the branch-specific clock rates under a relaxed clock model using the HMC machinery developed and described by Ji *et al.* [25]. In figure 2*b*, we demonstrate the relative efficiency of HMC over uMH in inferring branch-specific rates of phenotypic evolution on a large, fixed, 1536-tip HIV-1 tree. We report effective sample size (ESS) per second of BEAST runtime as a measure of efficiency since the ESS of a parameter is the number of effectively independent posterior samples in a given chain. We use HMC to investigate covariance between two traits associated with HIV virulence, the ‘gold standard viral load’ (GSVL) and CD4 cell count slope decline as they evolve on the fixed topology presented by Blanquart *et al.* [26]. Previous analyses of these data study trait covariation while assuming a time homogeneous, strict Brownian diffusion process guides trait evolution [27]. HMC makes it possible to learn about trait covariation under the more general relaxed random walk (RRW) model [28]. Fisher *et al.* [29] develop HMC machinery to make inference under the RRW. We find that the posterior mean estimate of covariance between GSVL and CD4 cell count slope decline is −0.163 with 95% highest posterior density (HPD) interval (−0.21, −0.11) and −0.151 ( −0.20, −0.10) under the strict Brownian diffusion and RRW, respectively. Similarly, 95% HPD intervals of individual variance estimates contain significant overlap between models as well. Overall, possible rate heterogeneity in the diffusion process does not influence the estimated covariation between GSVL and CD4 cell count slope decline. We, however, would not have known this without the availability of HMC to fit the richer model.

We additionally report the computational speed-up of estimating an internal node age using HMC versus uMH for the topologically fixed SARS-CoV-2 tree with 1000 full genome sequences (29 409 nt) of Lemey *et al.* [14]. We follow the model specifications of Lemey *et al.* [14] and model nucleotide evolution with an HKY85 with four category discrete-Γ site rate model and a coalescent tree prior for its induced prior on the node heights. We compare the accumulation of ESS per second of BEAST runtime using both traditional uMH BEAST default proposals as suggested by the BEAST graphical user interface, BEAUti, and recent HMC inference machinery developed by Ji *et al.* [30]. We estimate the age of the B.1.177 clade to be 0.45 years with 95% HPD interval (0.40, 0.50) and we obtain this estimate within a couple of hours, accumulating 63 ESS 1000 s^−1^ using the HMC machinery. Alternatively, under uMH, we do not obtain reliable estimates as quickly and only accumulate ESS at a rate of 4.0 ESS 1000 s^−1^. In this particular case, using HMC is approximately 15 times faster and can save up to 93% of runtime. We depict the trace plots of the node age under both samplers in figure 2*c*.

## 4. Alternatives and complements to Markov chain Monte Carlo

### (a) Sequential Monte Carlo methods

One alternative to approximating the posterior via MCMC is to use sequential Monte Carlo (SMC) methods, sometimes referred to as particle filters. Here, a particle represents a set of parameters of the model that characterize the evolutionary process and the tree topology. SMC methods start with a population of particles drawn from some easy-to-explore and tractable distribution (e.g. the prior distribution) and use importance sampling techniques to iteratively transform the distribution of particles towards the target posterior distribution. Therefore, SMC methods generate a collection of iterative distributions that transform to the posterior distribution in their last iteration. There are two major directions of development for SMC-based Bayesian phylogenetic methods. The first allows the dimensions of the parameter space to vary between iterations [31,32]. As introduced in §2, this direction is particularly useful when data arrive in an online fashion such that the dimensions of the state space grow with increasing number of taxa on the phylogeny. The other direction keeps the dimensions of the parameter space constant [33]. Wang *et al.* [33] generate a series of posteriors of equal dimension that gradually anneal towards the target posterior allowing for early escape from basins of attraction. This direction sets up a framework for SMC-based methods to employ the proposals developed in MCMC-based Bayesian phylogenetic machinery. This latter flavour of SMC fits within the broader category of particle Markov chain Monte Carlo (pMCMC) methods that combine SMC with MCMC [34]. Combinatorial sequential Monte Carlo is one such pMCMC algorithm and combines SMC tree samples with MCMC parameter estimates [35,36]. See Bouchard-Côté *et al.* [37] for further discussion of SMC as both an alternative to and complement to MCMC. Although pMCMC may ease the implementation of such methods into existing popular Bayesian phylogenetic software, only custom phylogenetic SMC methods are currently available (e.g. [33,36]).

### (b) Optimization to approximate the posterior

Variational inference is also an alternative method to MCMC for approximating the posterior distribution. While MCMC approximates via sampling from the posterior, variational inference approximates by optimizing over a user-chosen family of densities. The task is to find the density within the specified family of densities that minimizes the distance (more specifically, the Kullback–Leibler (KL) divergence) to the posterior [38]. Direct implementation of this approach is infeasible since computing the KL divergence requires calculating the typically intractable phylogenetic marginal likelihood. In practice, instead of minimizing the KL divergence, one maximizes the evidence lower bound (ELBO). Maximizing the ELBO is equivalent to finding the density that maximizes the expected value of the log-likelihood while minimizing the KL divergence with the prior [38].

Bayesian phylogenetic applications of variational inference employ a parametric family of distributions. Under this paradigm, one assumes parameters of interest, e.g. branch lengths in a phylogeny, are the parameters of the parametric family. Zhang & Matsen IV [39] develop a general variational phylogenetic framework to guide exploration in tree space while Dang & Kishino [40] offer a framework to make inference on the CAT model of amino acid evolution [41]. While these examples of phylogenetic variational inference show promising speed-up over some MCMC methods in their applications, current variational assumptions restrictively assume independence across variational parameters and examples of performance with more than a modest number of taxa (more than 100) remain open for exploration. It also remains an unexplored question, to what extent Bayesian phylogenetic variational inference may complement existing phylogenetic MCMC machinery. One possible direction of future exploration may employ fast variational approximation to set more informed starting points for MCMC analyses.

## 5. Discussion

Scalable Bayesian phylogenetics is growing to accommodate the challenge of analysing increasingly large datasets under complex models. Some methods, e.g. adaptive MCMC, HMC, parallelized computing, and SMC are new additions to the Bayesian phylogenetic toolkit, yet these are not at all new tools. Bayesian phylogenetics has been late to adopt modern inference approaches. One might be inclined to ask ‘why?’ One probable reason is that under many phylogenetic models, model parameters grow faster than the observed data. For example, when one observation is added to the tree, the number of branches, and thus the number of branch lengths to estimate, increases by two. Compound this with the difficulty of integrating over uncertainty in the combinatorially complex phylogenetic tree space and the result is that many methods may work for small toy examples but fail when scaled to larger analyses.

While the techniques presented here may alleviate computational burden in a variety of analyses, we have not provided an exhaustive overview of all scalable Bayesian phylogenetic approaches and their implementations. For example, Altekar *et al.* [42] develop an alternative adaptive MCMC approach within a Bayesian framework that runs multiple MCMC chains in parallel. Each chain is run at a different ‘temperature’. This effectively flattens the posterior so that more proposals are accepted and local extrema are avoided. Müller & Bouckaert [43] provide an implementation in BEAST. Another approach that handles multi-modality is the nested sampling method of Russel *et al.* [44]. Nested sampling, in a way conceptually similar to tempering, begins with a wide prior that grows successively more constrained as the sampler proceeds. Here the aim is model selection by marginal likelihood comparison and multi-modal inference is a by-product. Additionally, there are many recent developments aimed to improve tree sampling. We encourage readers interested in learning about efficient tree proposals to see Rannala & Yang [45] for inference under the multispecies coalescent, and Höhna & Drummond [46] for a comparison of the tradeoffs between computationally expensive guided tree proposals and naive fast proposals.

## Data Availability

We provide reproducible instructions and open access to the BEAST XML files to recreate the analyses in this paper at: https://github.com/suchard-group/scalableBayesianPhylogenetics.
